# Supplementary material for: Participant-derived cell line transcriptomic analyses and mouse studies reveal a role for ZNF335 in plasma cholesterol statin response
Source: bioRxiv. 2023 Jun 15:2023.06.14.544860. Preprint. [Version 1] doi: 10.1101/2023.06.14.544860 (PMC10312755; doi:10.1101/2023.06.14.544860)
Supplement: 2 [file NIHPP2023.06.14.544860v1-supplement-2.pdf]

bioRxiv preprint doi: https://doi.org/10.1101/2023.06.14.544860; this version posted June 15, 2023. The copyright holder for this preprint (which was not certified by peer review) is the author/funder, who has granted bioRxiv a license to display the preprint in perpetuity. It is made available under aCC-BY 4.0 International license.

Supplementary Figures

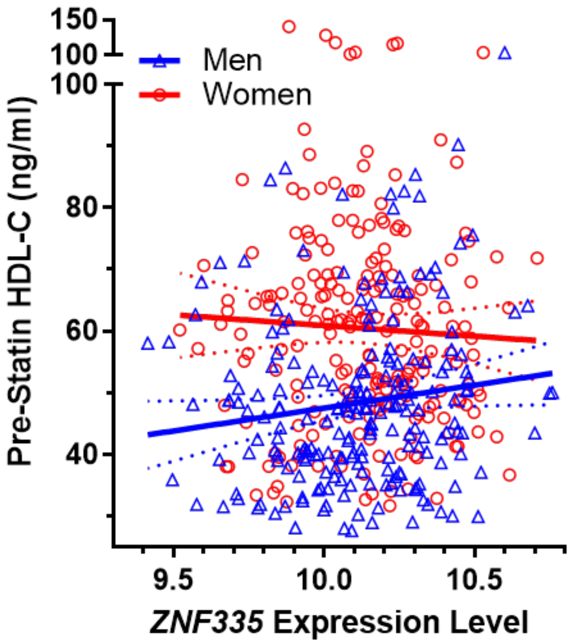

Figure S1. Correlation of pre-statin plasma HDL-cholesterol with *ZNF335* LCL gene expression levels from the corresponding donors split by sex (N=211 men, N=216 women).

bioRxiv preprint doi: https://doi.org/10.1101/2023.06.14.544860; this version posted June 15, 2023. The copyright holder for this preprint (which was not certified by peer review) is the author/funder, who has granted bioRxiv a license to display the preprint in perpetuity. It is made available under aCC-BY 4.0 International license.

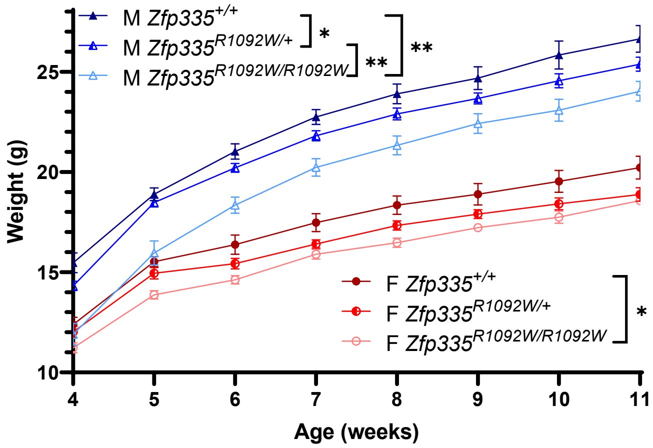

Figure S2. Mouse growth curves split by *Zfp335* genotype and sex. Adjusted p-values were calculated using the Compare Groups of Growth Curves (CGGC) method with 10,000 permutation tests. Male sample sizes were N=7, 19, and 7 and female sample sizes were N=7, 8, and 4 for wild type, heterozygotes, and homozygous Zfp335^{R1092W}, respectively. Values are mean ± SEM. *p<0.05 **p<0.01

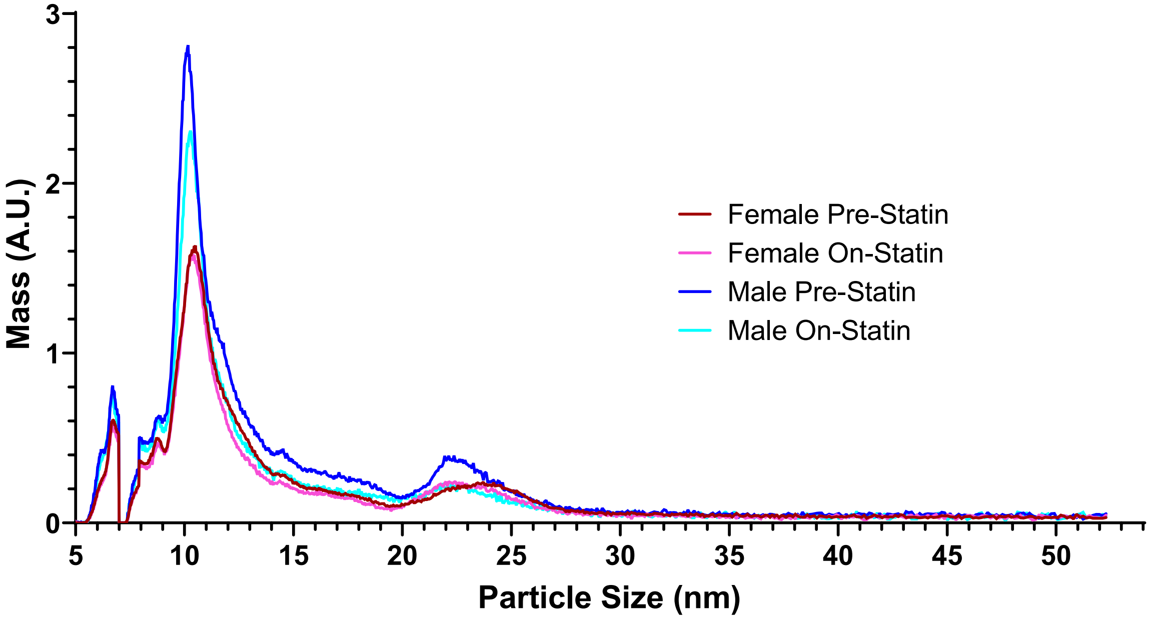

Figure S3. Statin-induced changes in wild-type N=5 male and N=11 female mouse lipoprotein profiles measured by ion mobility. Mouse profiles were measured before and after 4 weeks of simvastatin-containing diet. The size intervals designating the major lipoprotein subclasses are based on those defined in humans (29).

bioRxiv preprint doi: https://doi.org/10.1101/2023.06.14.544860; this version posted June 15, 2023. The copyright holder for this preprint (which was not certified by peer review) is the author/funder, who has granted bioRxiv a license to display the preprint in perpetuity. It is made available under aCC-BY 4.0 International license.

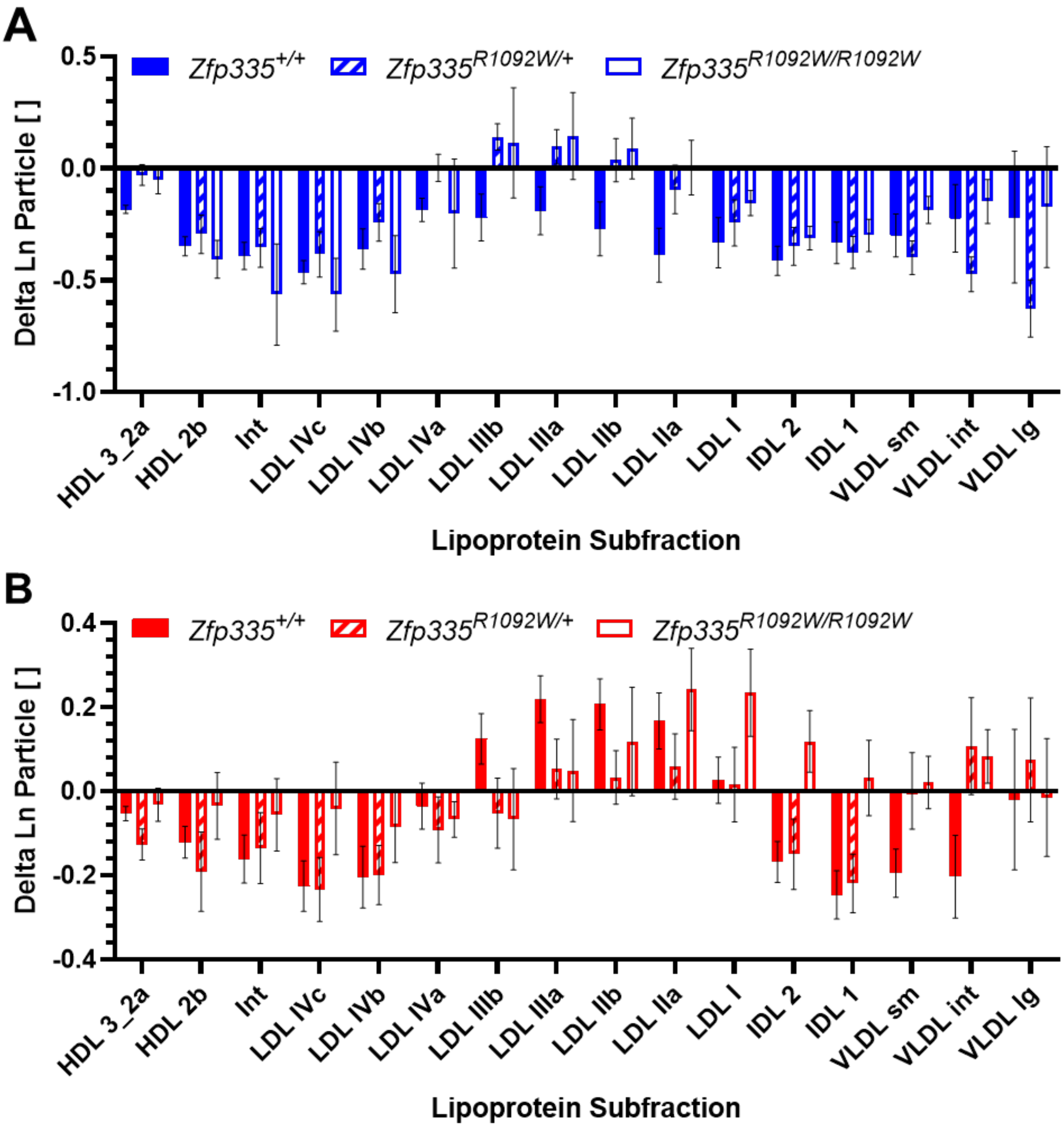

Figure S4. Statin-induced changes in (A) male and (B) female mouse plasma lipoprotein composition split by *Zfp335* genotype as measured by ion mobility. Male sample sizes were N=5, 12, and 5 and female sample sizes were N=11, 11, and 5 for wild type, heterozygotes, and homozygous Zfp335^{R1092W}, respectively. Values are mean ± SEM.

bioRxiv preprint doi: https://doi.org/10.1101/2023.06.14.544860; this version posted June 15, 2023. The copyright holder for this preprint (which was not certified by peer review) is the author/funder, who has granted bioRxiv a license to display the preprint in perpetuity. It is made available under aCC-BY 4.0 International license.

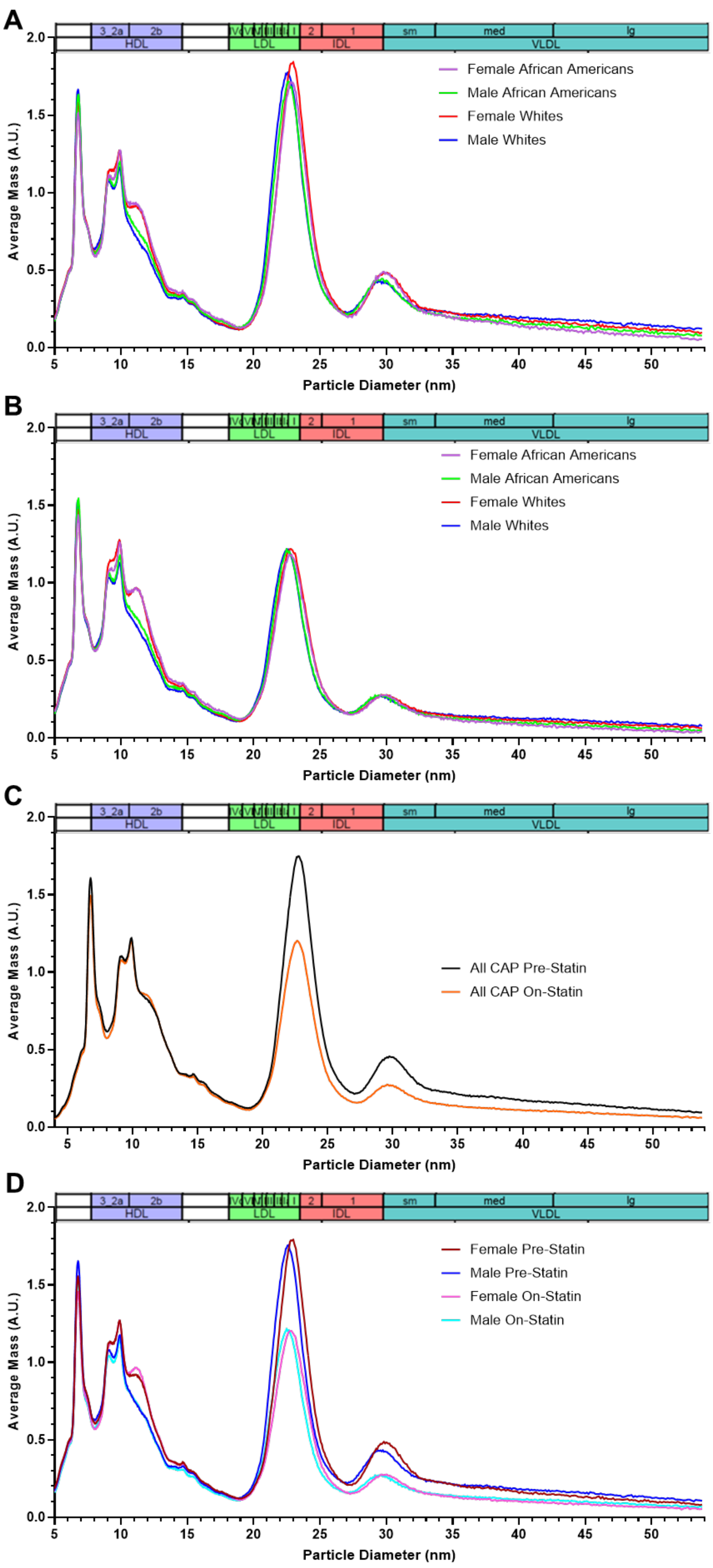

Figure S5. Ion mobility lipoprotein profiles for N=803 CAP participants before and after 6 weeks of 40 mg/day simvastatin treatment. Profiles shown are A) pre-statin split by sex and race/ethnicity B) on-statin split by sex and race/ethnicity C) pre- and on-statin for all and D) pre-statin and on-statin split by sex. N=142 for female African American, N=142 for male African American, N=243 for female white, and N=276 for male white participants.

bioRxiv preprint doi: https://doi.org/10.1101/2023.06.14.544860; this version posted June 15, 2023. The copyright holder for this preprint (which was not certified by peer review) is the author/funder, who has granted bioRxiv a license to display the preprint in perpetuity. It is made available under aCC-BY 4.0 International license.

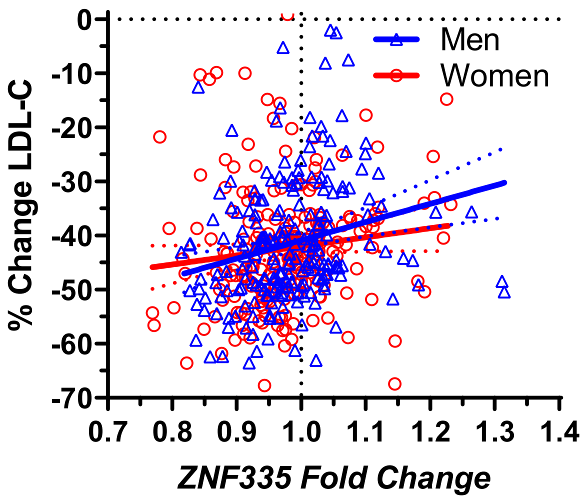

Figure S6. Correlation of plasma LDL-cholesterol statin response with *ZNF335* LCL gene expression statin response from the corresponding donors split by sex (N=211 men, N=216 women).
